# Radiomic analysis of postmortem lung changes: a PMCT-based approach for estimating the postmortem interval

**DOI:** 10.1007/s12024-025-01071-y

**Published:** 2025-08-27

**Authors:** Fabio De-Giorgio, Michele Guerreri, Luca Boldrini, Roberto Gatta, Eva Bergamin, Matteo Mancino, Evis Sala, Vincenzo L. Pascali

**Affiliations:** 1 Fondazione Policlinico Universitario A. Gemelli IRCCS, Rome, Italy; 2 Department of Healthcare Surveillance and Bioethics, Section of Legal Medicine, Università Cattolica del Sacro Cuore, Rome, Italy; 3 Department of Diagnostic Imaging and Oncological Radiotherapy, Fondazione Policlinico Universitario A. Gemelli IRCCS, Rome, Italy; 4 Section of Radiology, University Department of Radiological Sciences and Haematology, Università Cattolica del Sacro Cuore, Rome, Italy; 5 Dipartimento di Scienze Cliniche e mentali, Università degli Studi di Brescia, Brescia, Italy; 6 Department of Oncology, Lausanne University Hospital, Lausanne, Switzerland

**Keywords:** Autopsy, Computed tomography, Radiomics, Lung, Postmortem interval, Postmortem changes

## Abstract

This study presents an investigation of the potential of radiomic features extracted from postmortem computed tomography (PMCT) scans of the lungs to provide valuable insights into the postmortem interval (PMI), a crucial parameter in forensic medicine. Sequential PMCT scans were performed on 17 bodies with known times of death, ranging from 4 to 108 h postmortem. Radiomic features were extracted from the lungs, and a mixed-effects model, tailored for sequential data, was employed to assess the relationship between feature values and the PMI. Four model variants were tested to identify the most suitable functional form for describing this association. Several statistically significant trends between the PMI and radiomic features were observed, with twelve distinct features demonstrating selective relevance to postmortem changes in the lungs. Notably, cluster shade, a grey-level co-occurrence matrix (GLCM) feature, significantly decreased with the PMI, the median intensity increased over time, and the root mean squared feature values tended to decrease. The retained features included first-order statistical metrics, shape-based characteristics, and second-order texture attributes, which may reflect alterations such as gas formation and structural modifications within the lungs. This study highlights the potential of PMCT scan-based radiomics as a complementary tool to enhance existing postmortem interval estimation methods. These findings reinforce the role of quantitative imaging techniques in forensic investigations.

## Clinical trial number

N/A.

## Introduction

Determining the time since death is a fundamental task in forensic medicine. Accurately estimating the postmortem interval (PMI) helps law enforcement and significantly influences criminal investigations. This estimation relies on assessing various postmortem changes in the body [1]. However, the complexity of the task stems from multiple factors affecting these changes, including the cause of death; environmental conditions such as temperature and body location; and individual characteristics such as body mass, age, and sex.

To address this challenge, forensic experts have developed a variety of approaches, with the choice of method depending on the condition of the body [2]. In the early postmortem period, physical and biochemical processes—such as livor mortis, algor mortis, rigor mortis, and supravital reactivity—are commonly used [3]. As decomposition advances, techniques such as forensic entomology and the degradation of skeletal muscle proteins become more informative [4]. The continuous refinement of methods, incorporating new parameters and techniques, aims to improve the accuracy and reliability of PMI estimation [5, 6]. Consequently, research into novel approaches remains an active and evolving field [7, 8, 9].

Imaging techniques have long played a role in forensic investigations, often complementing or even replacing traditional autopsies [10, 11]. Among these methods, postmortem computed tomography (PMCT) has emerged as a preferred method because of its noninvasiveness and ability to provide high-contrast, high-resolution images [12, 13, 14]. PMCT enables detailed visualization of postmortem changes across various organs and tissues [15, 16, 17, 18, 19]. The lungs, in particular, undergo notable postmortem modifications that are detectable via PMCT and could serve as useful markers for PMI estimation. For example, the lungs show an intensity attenuation gradient, with areas of greater opacity localized in gravity-dependent regions [18]. With time, the increase in lung congestion and postmortem “oedema” reflects an increase in the extent and density of the attenuation gradient, up to a consolidation point [19], observed between 15 h and 38 h after death [20]. Moreover, the volume of the pleural space, the decreased aerated lung volume and the fraction of fluid in the airways are positively correlated with the PMI [21, 22]. However, despite the wealth of information provided by PMCT and other imaging modalities, a standardized method for leveraging these data in PMI estimation has yet to be established. One of the primary challenges lies in the lack of quantitative assessment.

Advancements in computational power, image processing, and data storage have facilitated the integration of machine learning (ML) algorithms into medical imaging analysis [23]. Radiomics, a cutting-edge approach in this field, operates on the principle that medical images contain vast amounts of data beyond what is visually apparent [24]. By extracting and analysing quantitative features from medical images, radiomics enables the identification of patterns and relationships that were previously inaccessible. While radiomics has been widely applied in various medical imaging fields [25, 26, 27, 28, 29], its use in postmortem imaging remains relatively unexplored [30, 31, 32, 33, 34, 35].

A recent study by Klontzas et al. [31] demonstrated the feasibility of using radiomics for PMI estimation on the basis of PMCT data from the liver and pancreas. Their approach successfully differentiated between early (< 12 h) and late (> 12 h) PMI. However, their study was based on cross-sectional data, meaning that each subject was analysed at a single point in time. While cross-sectional datasets are valuable for identifying correlations and constructing predictive models, they fall short in capturing the temporal progression of postmortem changes. Understanding how radiomic features evolve over time is crucial for improving the accuracy of PMI estimation.

Longitudinal studies, which involve repeated measurements from the same subjects over time, offer significant advantages over cross-sectional studies. By tracking changes within individuals, longitudinal data provide insights into the dynamics of postmortem transformations, allow for the identification of subject-specific patterns, and help control for confounding variables, leading to more robust and reliable findings.

This study aimed to explore the potential of radiomics for PMI estimation by utilizing longitudinal data instead of cross-sectional data. By repeatedly analysing the same subjects over time, we sought to gain a deeper understanding of how radiomic features evolve after death—insights that cross-sectional studies cannot provide. Specifically, we focused on the lungs, an organ that undergoes distinct postmortem changes and holds promise for PMI estimation. Through this longitudinal approach, we aimed to increase the precision and reliability of postmortem analyses, building upon existing knowledge while uncovering new dimensions of change.

The remainder of this manuscript is organized as follows: First, we describe the materials and methods used in the study, detailing the subject cohort, radiomics feature extraction, and statistical models applied to the data. Next, we present our results, discuss their implications, acknowledge the study’s limitations, and propose directions for future research.

## Materials and methods

### Subject recruitment

This study retrospectively included bodies that were brought to our Forensic Medicine Institute between May 2021 and May 2022. A total of 22 subjects were analysed, with an average age at the time of death of 57.7 years (age range: 18–86 years; SD = 21.4). Among them, 4 were female and 18 were male (Table 1). The inclusion criteria were a known time of death (verified by witnesses) and an age over 18 years. The exclusion criteria were the presence of clinically and/or radiologically significant lung pathologies and/or traumatic chest injuries. All 22 subjects meeting these criteria were included in the final analysis. All the bodies were transported to our Institute within 6 h of death. Upon arrival, the clothing was removed and the corpses were placed supine on the CT table in the CT room, with arms at their sides and wrapped in body bags. The corpses remained in this position throughout the procedure and remained in the CT room for the entire series acquisition in a controlled environment with a temperature of 18–20 °C and an air humidity of 49%.

**Table 1:** A comprehensive list of the studied cases, including baseline parameters (sex, age), cause of death, and postmortem interval (PMI) at the first and last postmortem computed tomography (PMCT) scans

Case	Sex	Age	Cause of death	PMI at first PMCT scan (min)	PMI at last PMCT scan (min)
**1**	**F**	**78**	Sudden cardiac death	411	1143
**2**	**M**	**75**	Head trauma	1480	4241
**3**	**M**	**15**	Hanging	782	2138
**4**	**M**	**83**	Sudden cardiac death	1585	3026
**5**	**M**	**84**	Sudden cardiac death	480	656
**6**	**M**	**58**	Sudden cardiac death	971	6534
**7**	**M**	**86**	Sudden cardiac death	283	2569
**8**	**M**	**75**	Sudden cardiac death	329	1159
**9**	**M**	**55**	Sudden cardiac death	340	550
**10**	**M**	**61**	Sudden cardiac death	251	683
**11**	**M**	**62**	Sudden cardiac death	1292	4412
**12**	**F**	**50**	Hanging	450	3000
**13**	**F**	**64**	Head trauma	1800	2907
**14**	**F**	**39**	Sepsis	565	1460
**15**	**M**	**51**	Hanging	1439	3447
**16**	**M**	**28**	Hanging	509	1819
**17**	**M**	**49**	Polytrauma	1017	2199
**18**	**M**	**43**	Head trauma	715	838
**19**	**M**	**53**	Sudden cardiac death	333	5697
**20**	**M**	**78**	Sudden cardiac death	431	592
**21**	**M**	**44**	Acute cocaine intoxication	1577	1577
**22**	**M**	**49**	Polytrauma	723	2122

Given that the bodies were under the jurisdiction of the Judicial Authority and thus subject to time constraints, organizational factors resulted in each corpse undergoing a variable number of sequential CT scans before autopsy, totalling 113 CT scans. On average, each subject underwent 5.4 scans (range = 3–16, SD = 3.5), with acquisitions performed at different PMIs. The average PMI acquisition time was 26.9 h postmortem (PMI range = 4.1–108.9 h, SD = 23.3). The average PMI at which the first scan was performed was 14.0 h postmortem (first scan PMI range = 4.2–30.9, SD = 8.6). All CT examinations were conducted using a Somatom Scope 16-slice CT scanner (Siemens Healthineers Italia) with the following parameters: 130 kV, 150 mA, 2.4 mm slice thickness, and H31S chest-district kernel reconstruction. Full-body CT scans were acquired from the skull vertex to beyond the feet (1.5 mm reconstructions). No contrast agent was used. No specific image preprocessing, such as external denoising or intensity normalization, was applied prior to feature extraction. Scans were evaluated by a senior radiologist for reliability and quality and were repeated in the case of artefacts. Following CT analysis, all the corpses underwent autopsy and standard histological/toxicological analyses. All investigations, including PMCT examination, autopsy, and histological/toxicological analyses, were authorized by the Judicial Authority.

### Image segmentation

**Fig. 1:**
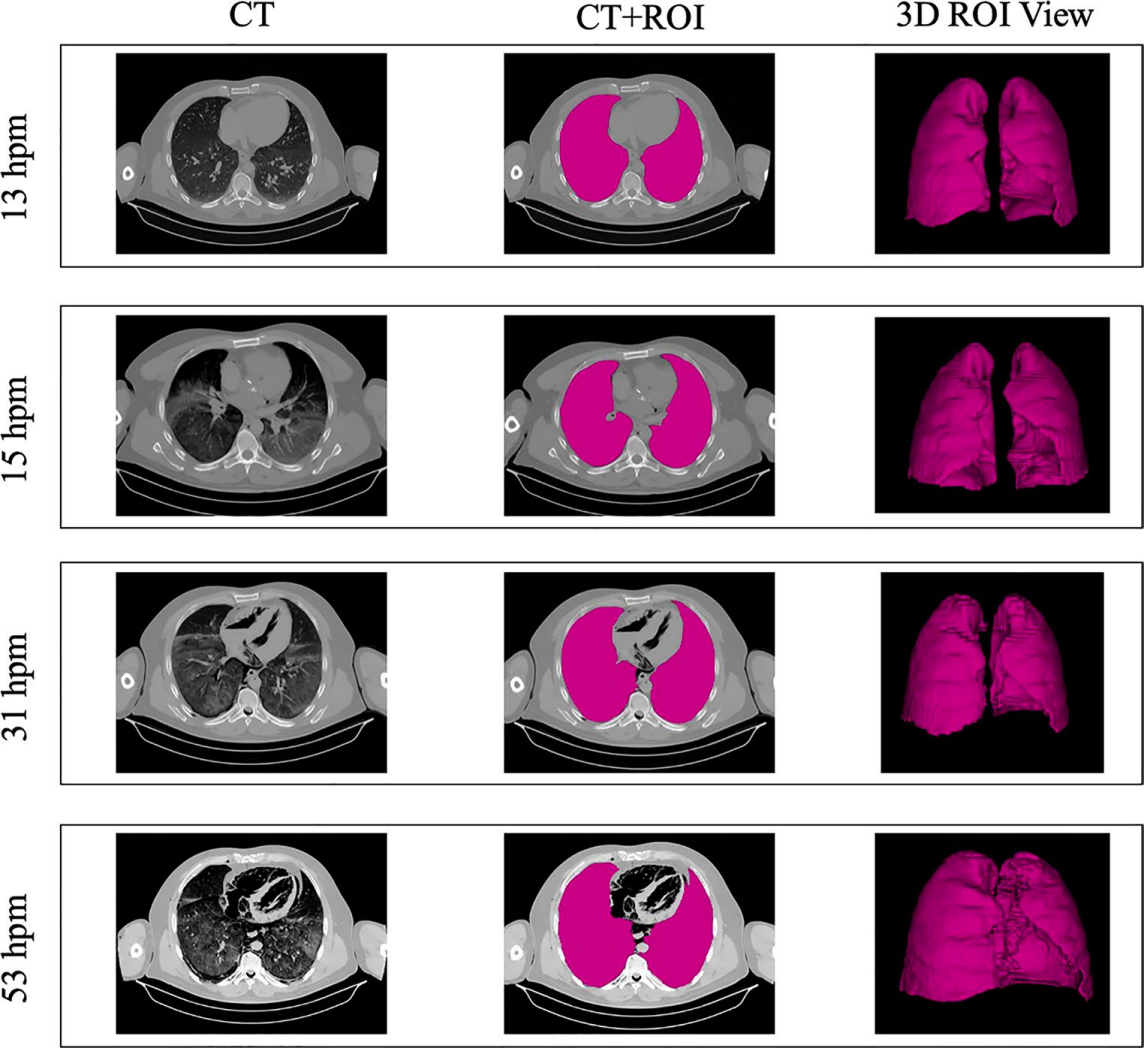
Lung segmentation and postmortem changes. Representative axial CT images (left), corresponding segmented lung regions (middle), and 3D lung renderings (right) acquired sequentially at distinct postmortem intervals (13, 15, 31, and 53 hpm) from the same subject, illustrating progressive postmortem changes

An experienced radiologist imported the PMCT series of each subject in DICOM format for analysis via the 3D Slicer platform (https://www.slicer.org), v5.4.0 [36]. A region of interest (ROI) was manually contoured for each subject’s lungs at multiple time points (Fig. 1). Segmentation was independently performed for each timepoint, without transferring or adapting contours from previous scans. This choice ensured that anatomical changes occurring over time were accurately captured during each acquisition. No scans were excluded because of motion artefacts, image quality issues, or segmentation failure. All three available views (axial, coronal, and sagittal) were considered. The ROIs were then exported into an RTSTRUCT file via the SlicerRT plug-in (v4.1.1).

### Radiomics feature extraction

The IBSI-compliant software MODDICOM, implemented in R (v4.3.0), was used to extract radiomic features from the PMCT images. A total of 117 features were extracted from the lung ROI, including first-order statistical features (21), shape-based 3D features (14), and second-order statistical features (82). The latter were further categorized into grey level co-occurrence matrix (GLCM) features (50) and grey level size zone matrix (GLSZM) features (32). GLCM and GLSZM features were computed in both 2D (averaged across slices) and 2.5D, which integrates information over multiple slices [37].

### Statistical methods

To assess whether any extracted radiomic features could serve as PMI predictors, we analysed their correlation with the PMI. Previous studies examining how lung density and volume change postmortem [38] have evaluated these relationships individually for each subject. In contrast, our study employed a mixed-effects model to account for the hierarchical structure of sequential scans, effectively managing multiple observations nested within each subject. The mixed model was defined as follows:

feature ~ f(PMI) + (1 | subject) (1).

Here, the dependent variable represents an extracted radiomic feature, whereas the independent variable is a function of the PMI, denoted as f(PMI). The model includes a random intercept, treating the slope as a fixed effect. This approach accounts for within-subject variability and accommodates repeated measures. A flexible function of the PMI allows for nonlinear modelling, such as f(PMI) = ln(PMI). Statistical analyses were conducted using R (v4.1.0), with model fitting performed via the *lmer* function from the lme4 package (v1.1-35.3).

### Experiments

Our objective was to identify radiomic features strongly associated with the PMI that could serve as potential predictors. For each extracted feature, we tested four model variants differing in their function of PMI: f(PMI) = PMI, PMI², PMI¹/², and ln(PMI). Statistical significance was assessed using a t test to determine whether fixed effect coefficients were significantly different from zero. To adjust for multiple comparisons, p values were corrected using the false discovery rate (FDR) method, with a significance threshold of *p* < 0.05.

To determine the best-fitting model for each feature, we evaluated model performance on the basis of log-likelihood scores. Features with significant PMI associations underwent additional selection to remove those highly correlated with one another. Pairwise correlation was assessed, and if the correlation coefficient exceeded 0.7, the feature with the weaker PMI association (higher p value) was discarded.

## Results

A total of 468 models (117 features × 4 model variants) were fitted to the data. We found a statistically significant association between radiomic features and the PMI for 169 of these models. There were 57 different features that showed a statistically significant association for at least one of the model variants. Among these features, 12 were first-order statistical features, 4 were shape-based features, 32 were GLCM features, and 9 were GLSZM features. Table 2 reports the results of the feature selection, in which we discarded features with high correlations, retaining only those that exhibit exclusive information about the processes that the lungs undergo as the PMI increases. Of the 57 features found in the previous step, only 12 were retained after the feature selection step. Among these features, 5 were first-order statistical features, 4 were shape-based features, 3 were GLCM features, and no GLSZM features were selected.

Among the retained features, those with the strongest association with the PMI in terms of the p value were the cluster shade, a GLCM feature, which decreased with the PMI (*p* < 3e-12); the median intensity value, which increased with the PMI (*p* < 3e-11); the root mean squared feature value, a first-order statistical feature, which decreased with the PMI (*p* < 2e-11); and the 2.5D energy, a GLCM feature, which was found to decrease (*p* < 5e-5), whereas the shape-based feature centre of mass (COM) was found to increase (*p* < 1e-4).

For each of the selected features, Table 2 also reports the model variant that provided the best fit performance, i.e., the model variant with the lowest AIC. For all the features, a logarithmic relationship between the feature values and the PMI best described the data.

**Fig. 2:**
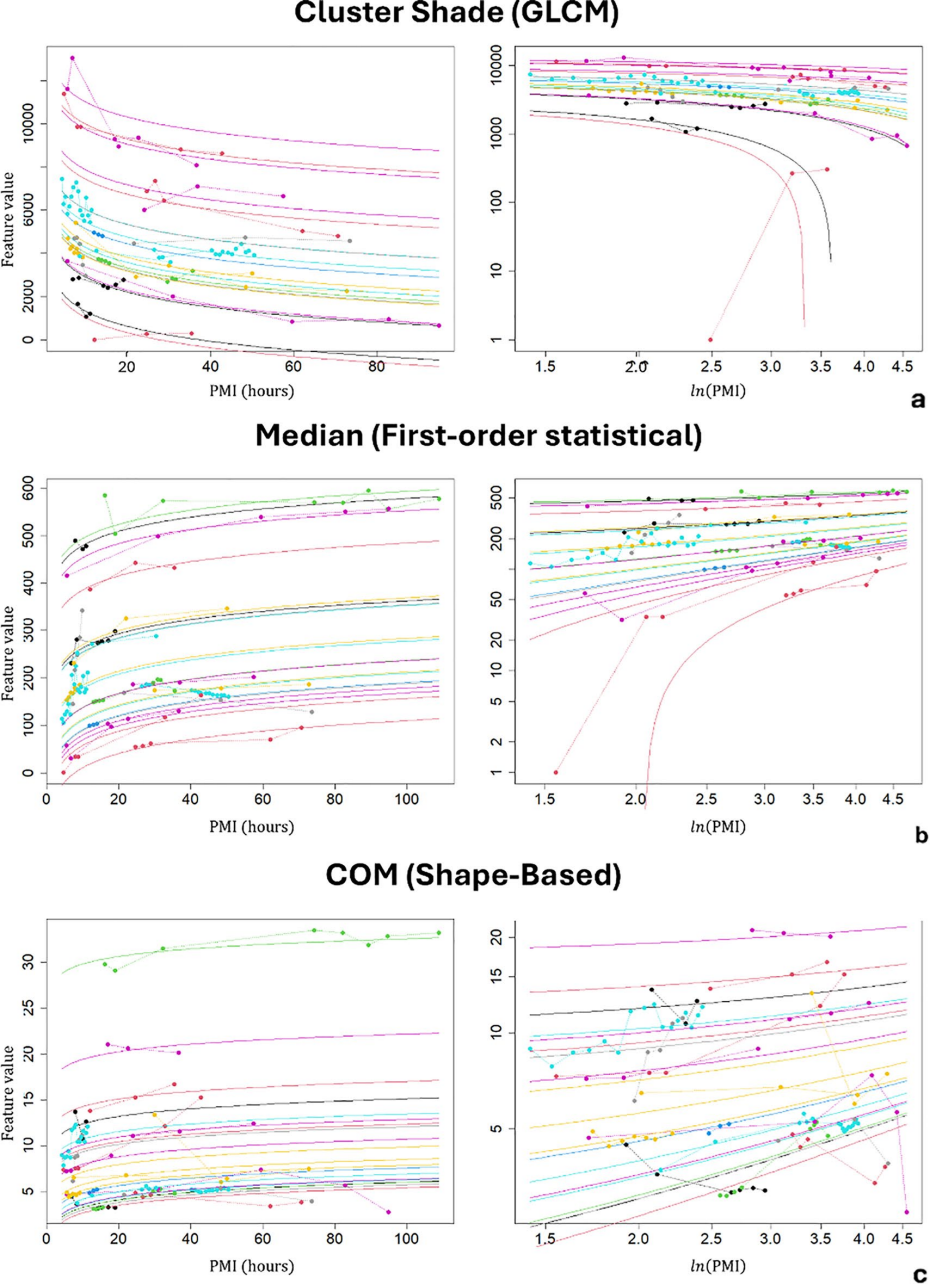
Radiomic features vs. PMI plots. The figure shows the associations between several representative radiomic features extracted from the lungs and the postmortem interval (PMI), expressed in hours. Three features are reported: cluster shade, a second-order statistical feature belonging to the GLCM group. The lung median intensity value is a first-order statistical feature. The centre of mass (COM) is a shape-based feature. The features are exemplars of the different feature classes. For each feature, we report two plots: on the left, we plot the feature value as a function of the PMI to show the different trends; on the right, we report the feature values as a function of f(PMI), where f(x) is equal to ln(x). Furthermore, in this latter plot, we use log-scale axes to improve the data readability. In each plot, the data from the same subjects have the same colours and are connected by dashed lines. The solid lines represent the fitted models. Different lines correspond to the different random effects estimated by the model, while the slope (the fixed effect) is shared by the subjects

Figure 2 presents three examples that illustrate how feature values are plotted against the PMI. Each example highlights a different class of feature that was significantly associated with the PMI. In these plots, data points are depicted as dots, with points from the same subject sharing the same colour and connected by dashed lines. Additionally, the plots feature fitted models represented by solid lines. The number of solid lines corresponds to the number of subjects included in the study, with each line consistently coloured according to the respective subject. These plots demonstrate how the model captures intersubject variability through random effects (model intercepts), whereas the overall variation in feature values as a function of the PMI is explained by fixed effects (the slopes of the curves).

## Discussion

This study quantitatively analysed postmortem lung changes detected through PMCT by extracting radiomic features that capture intensity, texture, and shape alterations over time. Our findings confirm that PMCT-based radiomics holds promise for enhancing PMI estimation by identifying features strongly correlated with PMI.

After death, the lungs undergo a series of progressive anatomical and physiological changes that significantly alter their appearance on CT scans. Hypostasis occurs in the lung postmortem and can be identified as a hyperdense gravity-dependent gradient that is bilateral and symmetrical [19, 22]. Furthermore, deposits of endotracheal fluid are also common [22]. Over time, this gradient intensifies as pulmonary congestion and postmortem “oedema” progress, eventually leading to widespread consolidation, typically observed between 15 and 38 h after death [19, 20]. Concurrently, measurable anatomical changes such as reduced lung volume and increased pleural space volume have been shown to correlate positively with advancing PMI [21, 22]. These progressive postmortem changes can be detected by CT imaging, making the lungs a particularly suitable target for radiomic analysis, as their evolving structural and textural patterns can be quantitatively captured through advanced image-based features.

In this work, a total of 468 models were tested, and we identified statistically significant associations between the PMI and radiomic features in 169 models. After feature selection, 12 features were retained, representing first-order statistics, shape-based descriptors, and texture-related features from the GLCM. Notably, no GLSZM features were selected, suggesting that PMI-related changes in the lungs are better captured by intensity distribution, structural alterations, and localized texture variations than by larger-scale zone uniformity patterns [18, 19].

Among the retained features, cluster shade (a GLCM feature) showed the strongest association with the PMI, decreasing as the PMI increased. This feature quantifies asymmetry in texture patterns, and its decline may reflect the progressive homogenization of lung tissue texture due to fluid accumulation, gas formation, or the breakdown of pulmonary microstructures. Another key feature, the median intensity value, increased over time, suggesting a shift in voxel intensity that may correspond to the accumulation of intrapulmonary gas, changes in tissue density, or the redistribution of postmortem fluids [18, 19, 20, 21, 22, 39].

The root mean squared (RMS) intensity, a first-order statistical feature, also significantly decreased over time. The RMS is sensitive to variations in intensity distribution, and its decrease could indicate a reduction in contrast between the lung parenchyma and surrounding structures due to postmortem consolidation or collapse. Similarly, the 2.5D energy feature, another GLCM-based metric, was found to decrease as the PMI increased. This metric reflects the overall intensity uniformity within an image; its decline may suggest progressive tissue decomposition and the loss of structural integrity.

In contrast, entropy, which quantifies the randomness or complexity of intensity patterns, increases with the PMI. This increase may reflect the disruption of the normal anatomical structure, the mixing of air and fluid densities, and increased voxel-level heterogeneity due to decomposition-related processes.

Shape-based features also exhibited significant changes over time. The centre of mass (COM) increased as the PMI progressed, which may be related to the redistribution of the lung volume due to collapse, fluid shifts, or putrefactive gas expansion [21, 22, 39].

Notably, a logarithmic function best described the relationship between the PMI and all selected features, suggesting that the rate of postmortem changes slows down as the PMI increases, possibly due to the stabilization of gas accumulation and tissue breakdown processes.

Our analysis also highlighted intersubject variability, captured through random effects in our models. This variability underscores the complex interplay between individual anatomical differences, environmental factors, and the specific mechanisms governing postmortem lung changes. The use of longitudinal data provided critical insights into these dynamics, allowing us to track feature evolution within individual subjects rather than relying on cross-sectional comparisons.

A previous study by Klontzas et al. [31] demonstrated the feasibility of using radiomic features from the liver and pancreas to predict the PMI using cross-sectional data. Our study builds upon this concept by focusing on the lungs and adopting a longitudinal approach. While we did not construct a predictive model, our findings highlight radiomic features that could serve as robust markers for PMI estimation. Future studies could leverage these features to develop predictive models similar to those proposed by Klontzas et al. while also incorporating longitudinal elements to increase accuracy.

Standardizing imaging protocols remains a critical step for ensuring reproducibility in forensic radiomics. The implementation of uniform scanning procedures across forensic centres would facilitate the validation of our findings and improve the consistency of PMI estimations across different populations.

One limitation of this study is the assumption that all the subjects exhibited similar rates of change in radiomic features over time. Owing to the relatively small sample size, we treated feature variation as a fixed effect. However, larger studies with more frequent sampling could incorporate random slope effects, allowing for a more individualized assessment of postmortem lung changes. Additionally, the sample lacked diversity in sex, with only 4 women among the 22 cases, which may have introduced bias in the results.

## Conclusions

This study presents a quantitative analysis of postmortem lung modifications using PMCT-based radiomics. Our findings demonstrate that several radiomic features are strongly associated with the PMI, suggesting their potential utility in refining PMI estimation techniques.

Radiomics offers several advantages in this context: its quantitative nature enhances objectivity compared with traditional visual assessment methods, reducing subjective bias in forensic investigations. Additionally, radiomics enables the detection of subtle textural and structural changes in lung tissue that may not be visible through conventional imaging analysis. The variety of extracted features—spanning shape, intensity, and texture—provides a comprehensive characterization of postmortem pulmonary changes, which could contribute to more accurate PMI estimation models.

Our results support the integration of PMCT-based radiomics into forensic workflows. By leveraging standardized radiomic biomarkers, future studies could refine PMI estimation techniques, improve forensic accuracy, and enhance the reliability of postmortem investigations.

However, a major limitation of many radiomic-based approaches lies in their potential lack of repeatability and generalizability across different imaging protocols, scanners, and populations. To address this, future research should aim to validate these findings through multicentre studies using harmonized imaging and analysis protocols. These studies would provide crucial evidence on the robustness and reproducibility of radiomic biomarkers in diverse forensic settings. Additionally, incorporating automated segmentation tools could improve standardization and reduce interoperator variability. Radiomic analysis could also be integrated with other forensic indicators, such as biochemical or microbiological markers, to develop more comprehensive and reliable models for PMI estimation.

In summary, while our results are promising, further validation on larger and more heterogeneous datasets is essential to demonstrate the broader applicability of radiomics in postmortem imaging and to establish its role as a standardized tool in forensic pathology.

**Table 2:** Feature selection results. The features retained after the feature selection process are reported in the table. In addition, the table reports the class type to which the feature belongs (first-level statistical, second-level statistical, shape-based), the model variant that gave the best results in terms of fit performance (simple linear, logarithmic, square-root), the p value obtained to test whether the model’s fixed effect was different from zero, the fit performance in terms of the log-likelihood (log-like), the conditional R2 of the model and the fixed effect obtained from the fit.

Name		Feature Class	Model Variant	*p*-val	Log-like	Cond *R*^2^	Fixed Eff	Std Err
Clust. Shade	2nd Lev. Stat.		Log	2.32E-12	-890.87	0.98	-1.02E + 03	1.11E + 02
Median	1st Lev. Stat.		Log	2.73E-11	-553.86	0.97	4.31E + 01	5.02E + 00
RMS	1st Lev. Stat.		Log	1.87E-10	-512.00	0.97	-2.73E + 01	3.39E + 00
Energy (2.5D)	2nd Lev. Stat.		Log	4.07E-05	481.96	0.96	-1.76E-03	3.52E-04
**COM**	Shape-Based		Log	1.35E-04	-231.60	0.96	1.20E + 00	2.59E-01
10th Perc.	1st Lev. Stat.		Log	1.36E-04	-526.29	0.97	1.79E + 01	3.90E + 00
Joint Entr. (2.5D)	2nd Lev. Stat.		Log	3.19E-04	-12.25	0.92	1.58E-01	3.64E-02
Kurtosis	1st Lev. Stat.		Log	4.72E-03	-97.42	0.93	-2.80E-01	7.94E-02
L-minor	Shape-Based		Log	5.16E-03	-354.90	0.98	-2.74E + 00	7.83E-01
Entropy	1st Lev. Stat.		Log	9.28E-03	56.55	0.72	6.85E-02	2.09E-02
L-major	Shape-Based		Log	1.14E-02	-349.58	0.98	-2.39E + 00	7.42E-01
Compactness 2	Shape-Based		Log	1.31E-02	334.05	0.88	-4.78E-03	1.52E-03

Clust. Shade = cluster shade; rms = root mean square intensity value; com = centre of mass; 10th percentile = 10th percentile; joint. Entr. (2.5D) = 2.5D joint entropy

## Key Points

1. To investigate whether radiomic features extracted from PMCT scans can provide meaningful information for estimating the PMI.
2. Radiomic features from PMCT lung scans show potential for PMI estimation.
3. Twelve radiomic features showed significant trends, linking the PMI to postmortem changes in the lung.
4. PMCT-based radiomics may enhance forensic methods for estimating the time since death.

## Data Availability

N/A.

## Code availability

N/A.

## Declarations

Not needed.

## References

[1] Madea B. Estimation of the time since death. 4th ed. Boca Raton (FL): CRC; 2023.

[2] Henssge C, Madea B. Estimation of the time since death in the early post-mortem period. Forensic Sci Int. 2004;144(2–3):167–75. 15364387 10.1016/j.forsciint.2004.04.051

[3] Bate-Smith EC, Bendall JR. Rigor mortis and adenosinetriphosphate. J Physiol. 1947;106(2):177–85. 20249085

[4] Amendt J, Richards CS, Campobasso CP, Zehner R, Hall MJR. Forensic entomology: applications and limitations. Forensic Sci Med Pathol. 2011;7(4):379–92. 21213072 10.1007/s12024-010-9209-2

[5] Henssge C. Death time estimation in case work. I. The rectal temperature time of death nomogram. Forensic Sci Int. 1988;38(3–4):209–36. Available from: https://www.sciencedirect.com/science/article/abs/pii/0379073888901685 10.1016/0379-0738(88)90168-5 3192144

[6] Henssge C, Althaus L, Bolt J, Freislederer A, Haffner HT, Henssge CA, et al. Experiences with a compound method for estimating the time since death. Int J Legal Med. 2000;113(6):320–31. 11100426 10.1007/s004149900090

[7] Thakral S, Purohit P, Mishra R, Gupta V, Setia P. The impact of RNA stability and degradation in different tissues to the determination of post-mortem interval: a systematic review. Forensic Sci Int. 2023;349:111772. 37450949 10.1016/j.forsciint.2023.111772

[8] De-Giorgio F, Ciasca G, D’Amico R, Trombatore P, D’Angelo A, Rinaldi P, et al. An evaluation of the objectivity and reproducibility of shear wave elastography in estimating the post-mortem interval: a tissue Biomechanical perspective. Int J Legal Med. 2020;134(5):1939–48. 32676888 10.1007/s00414-020-02370-5

[9] De-Giorgio F, Nardini M, Foti F, Minelli E, Papi M, d’Aloja E, et al. A novel method for post-mortem interval Estimation based on tissue nano-mechanics. Int J Legal Med. 2019;133(4):1133–9. 30919038 10.1007/s00414-019-02034-z

[10] Brogdon BG. Scope of forensic radiology. Crit Rev Diagn Imaging. 2000;41(1):43–67. 10710698

[11] Eckert WG, Garland N. The history of the forensic application in radiology. Am J Forensic Med Pathol. 1984;5(1):53–6. 6369961 10.1097/00000433-198403000-00010

[12] Krantz P, Holtås S. Postmortem computed tomography in a diving fatality. J Comput Assist Tomogr. 1983;7(1):132–4. 6826832 10.1097/00004728-198302000-00024

[13] Donchin Y, Rivkind AI, Bar-Ziv J, Hiss J, Almog J, Drescher M. Utility of postmortem computed tomography in trauma victims. J Trauma Inj Infect Crit Care. 1994;37(4):552–6. 10.1097/00005373-199410000-00006 7932884

[14] Thali MJ, Yen K, Schweitzer W, et al. Virtopsy, a new imaging horizon in forensic pathology: virtual autopsy by postmortem multislice computed tomography (MSCT) and magnetic resonance imaging (MRI)—a feasibility study. J Forensic Sci. 2003;48(2):386–403. 12665000

[15] Christe A, Flach P, Ross S, et al. Clinical radiology and postmortem imaging (Virtopsy) are not the same: specific and unspecific postmortem signs. Leg Med (Tokyo). 2010. 10.1016/j.legalmed.2010.05.005. 20630787 10.1016/j.legalmed.2010.05.005

[16] Levy AD, Harcke HT, Mallak CT. Postmortem imaging: MDCT features of postmortem change and decomposition. Am J Forensic Med Pathol. 2010;31(1):12–7. 20010292 10.1097/PAF.0b013e3181c65e1a

[17] Okuma H, Gonoi W, Ishida M, Shirota G, Shintani Y, Abe H, et al. Comparison of Attenuation of striated muscle between postmortem and antemortem computed tomography: results of a longitudinal study. PLoS ONE. 2014;9(11):e111457. 25365255 10.1371/journal.pone.0111457 PMC4218726

[18] Shiotani S, Kohno M, Ohashi N, Yamazaki K, Nakayama H, Watanabe K, et al. Non-traumatic postmortem computed tomographic (PMCT) findings of the lung. Forensic Sci Int. 2004;139(1):39–48. 14687772 10.1016/j.forsciint.2003.09.016

[19] Shiotani S, Kobayashi T, Hayakawa H, Kikuchi K, Kohno M. Postmortem pulmonary edema: a comparison between immediate and delayed postmortem computed tomography. Leg Med (Tokyo). 2011;13(3):151–5. Available from: https://pubmed.ncbi.nlm.nih.gov/21315646/10.1016/j.legalmed.2010.12.008 21315646

[20] Michiue T, Ishikawa T, Oritani S, Kamikodai Y, Tsuda K, Okazaki S, et al. Forensic pathological evaluation of postmortem pulmonary CT high-density areas in serial autopsy cases of sudden cardiac death. Forensic Sci Int. 2013;232(1–3):199–205. 24053881 10.1016/j.forsciint.2013.07.025

[21] Hyodoh H, Shimizu J, Watanabe S, Okazaki S, Mizuo K, Inoue H. Time-related course of pleural space fluid collection and pulmonary aeration on postmortem computed tomography (PMCT). Leg Med (Tokyo). 2015;17(4):221–5. 25657038 10.1016/j.legalmed.2015.01.002

[22] Ishida M, Gonoi W, Hagiwara K, Okuma H, Shintani Y, Abe H, et al. Fluid in the airway of nontraumatic death on postmortem computed tomography. Am J Forensic Med Pathol. 2014;35(2):113–7. 24781399 10.1097/PAF.0000000000000083

[23] Lambin P, Rios-Velazquez E, Leijenaar R, Carvalho S, van Stiphout RGPM, Granton P, et al. Radiomics: extracting more information from medical images using advanced feature analysis. Eur J Cancer. 2012;48(4):441–6. 22257792 10.1016/j.ejca.2011.11.036 PMC4533986

[24] Gillies RJ, Kinahan PE, Hricak H, Radiomics. Images are more than pictures, they are data. Radiology. 2016;278(2):563–77. 26579733 10.1148/radiol.2015151169 PMC4734157

[25] Nardone V, Boldrini L, Grassi R, Franceschini D, Morelli I, Becherini C et al. Radiomics in the setting of neoadjuvant radiotherapy: a new approach for tailored treatment. Cancers. 2021 [cited 2024 Jan 20];13(14):3590. Available from: https://www.mdpi.com/2072-6694/13/14/3590. 10.3390/cancers13143590 PMC8303203 34298803

[26] Calandrelli R, Boldrini L, Tran HE, Quinci V, Massimi L, Pilato F, et al. CT-based radiomics modeling for skull dysmorphology severity and surgical outcome prediction in children with isolated sagittal synostosis: a hypothesis-generating study. La Radiol Med. 2022;127(6):616–26. 10.1007/s11547-022-01493-6 PMC9130191 35538388

[27] Barnes H, Humphries SM, George PM, Assayag D, Glaspole I, Mackintosh JA, et al. Machine learning in radiology: the new frontier in interstitial lung diseases. Lancet Digit Health. 2023;5(1):e41–50. 36517410 10.1016/S2589-7500(22)00230-8

[28] Liang S, Ma J, Wang G, Shao J, Li J, Deng H et al. The application of artificial intelligence in the diagnosis and drug resistance prediction of pulmonary tuberculosis. Front Med. 2022;9. 10.3389/fmed.2022.935080 PMC9366014 35966878

[29] Giacobbe G, Granata V, Trovato P, Fusco R, Simonetti I, Muzio FD, et al. Gender medicine in clinical radiology practice. J Pers Med. 2023;13(2):223. 36836457 10.3390/jpm13020223 PMC9966684

[30] De-Giorgio F, Ciasca G, Fecondo G, Mazzini A, Di Santo R, De Spirito M, et al. Post mortem computed tomography Meets radiomics: a case series on fractal analysis of post mortem changes in the brain. Int J Legal Med. 2022;136(3):719–27. 35239030 10.1007/s00414-022-02801-5 PMC9005394

[31] Klontzas ME, Leventis D, Spanakis K, Karantanas AH, Kranioti EF. Post-mortem CT radiomics for the prediction of time since death. Eur Radiol. 2023;33(11):8387–95. 37329460 10.1007/s00330-023-09746-2

[32] Fan F, Liu H, Dai X, Liu G, Liu J, Deng X, et al. Automated bone age assessment from knee joint by integrating deep learning and MRI-based radiomics. Int J Legal Med. 2023;138(3):927–38. 38129687 10.1007/s00414-023-03148-1

[33] Michaud K, Grabherr S, Jackowski C, Bollmann MD, Doenz F, Mangin P. Postmortem imaging of sudden cardiac death. Int J Legal Med. 2014;128(1):127–37. 23322013 10.1007/s00414-013-0819-6

[34] Viero A, Biehler-Gomez L, Messina C, Cappella A, Giannoukos K, Viel G, et al. Utility of micro-CT for dating post-cranial fractures of known post-traumatic ages through 3D measurements of the trabecular inner morphology. Sci Rep. 2022;12(1):10543. 35732857 10.1038/s41598-022-14530-1 PMC9218115

[35] De-Giorgio F, Guerreri M, Gatta R, Bergamin E, De Vita V, Mancino M, et al. Exploring radiomic features of lateral cerebral ventricles in postmortem CT for postmortem interval Estimation. Int J Legal Med. 2024;139(2):667–77. 39702800 10.1007/s00414-024-03396-9

[36] Fedorov A, Beichel R, Kalpathy-Cramer J, Finet J, Fillion-Robin JC, Pujol S, et al. 3D slicer as an image computing platform for the quantitative imaging network. Magn Reson Imaging. 2012;30(9):1323–41. 22770690 10.1016/j.mri.2012.05.001 PMC3466397

[37] Zwanenburg A, Vallières M, Abdalah MA, Aerts HJWL, Andrearczyk V, Apte A, et al. The image biomarker standardization initiative: standardized quantitative radiomics for high-throughput image-based phenotyping. Radiology. 2020;295(2):328–38. 32154773 10.1148/radiol.2020191145 PMC7193906

[38] Hasegawa I, Shimizu A, Saito A, Suzuki H, Vogel H, Püschel K, et al. Evaluation of post-mortem lateral cerebral ventricle changes using sequential scans during post-mortem computed tomography. Int J Legal Med. 2016;130(5):1323–8. 27048214 10.1007/s00414-016-1327-2 PMC4976059

[39] Filograna L, Thali MJ. Post-mortem CT imaging of the lungs: pathological versus non-pathological findings. Radiol Med. 2017;122(12):902–8. 28836139 10.1007/s11547-017-0802-2

